# The adsorption of biomolecules to multi-walled carbon nanotubes is influenced by both pulmonary surfactant lipids and surface chemistry

**DOI:** 10.1186/1477-3155-8-31

**Published:** 2010-12-15

**Authors:** Michael Gasser, Barbara Rothen-Rutishauser, Harald F Krug, Peter Gehr, Mathias Nelle, Bing Yan, Peter Wick

**Affiliations:** 1 Empa, Swiss Federal Laboratories for Materials Science and Technology, Laboratory for Materials Biology Interactions, St. Gallen, Switzerland; 2 Institute of Anatomy, Division of Histology, University of Bern, Bern, Switzerland; 3 Division Neonatology, Department of Paediatrics, Inselspital and University of Bern, Bern, Switzerland; 4 Department of Chemical Biology and Therapeutics, St. Jude Children's Research Hospital, Memphis, TN 38105, USA and School of Chemistry and Chemical Engineering, Shandong University, Jinan, 250100, China

## Abstract

**Background:**

During production and processing of multi-walled carbon nanotubes (MWCNTs), they may be inhaled and may enter the pulmonary circulation. It is essential that interactions with involved body fluids like the pulmonary surfactant, the blood and others are investigated, particularly as these interactions could lead to coating of the tubes and may affect their chemical and physical characteristics. The aim of this study was to characterize the possible coatings of different functionalized MWCNTs in a cell free environment.

**Results:**

To simulate the first contact in the lung, the tubes were coated with pulmonary surfactant and subsequently bound lipids were characterized. The further coating in the blood circulation was simulated by incubating the tubes in blood plasma. MWCNTs were amino (NH_2_)- and carboxyl (-COOH)-modified, in order to investigate the influence on the bound lipid and protein patterns. It was shown that surfactant lipids bind unspecifically to different functionalized MWCNTs, in contrast to the blood plasma proteins which showed characteristic binding patterns. Patterns of bound surfactant lipids were altered after a subsequent incubation in blood plasma. In addition, it was found that bound plasma protein patterns were altered when MWCNTs were previously coated with pulmonary surfactant.

**Conclusions:**

A pulmonary surfactant coating and the functionalization of MWCNTs have both the potential to alter the MWCNTs blood plasma protein coating and to determine their properties and behaviour in biological systems.

## Background

Carbon nanotubes (CNTs), discovered in the early 1990's [1], have been brought into focus due to their outstanding mechanical, electronic, optical and magnetic properties. In a rapidly growing field, numerous new applications have been developed and the need for CNTs has reached industrial production scale [2]. However, the exposure risks during the processing and production of CNTs has also increased substantially. It is known from studies with nano-sized particles [3] and CNTs [4,5] that exposure by inhalation is the primary exposure route for humans.

Due to their size and shape, inhaled CNTs may reach the alveolar region [6,7]. Upon deposition, they come in initial contact with the pulmonary surfactant, which is located at the air-liquid interface. Surfactant contains 85-90% phospholipids [8] and has an essential function during breathing by reducing the surface tension [9]. Adsorption of pulmonary surfactant phospholipids was shown on nano-sized gold particles [10] and on carbon black nano-sized particles [11]. In contrast, interactions of CNTs with complex mixtures of pulmonary surfactant lipids have not been studied in detail so far.

By wetting forces, nano-sized particles are displaced into the hypophase [12-14] and may be translocated across the air-blood tissue barrier by crossing the epithelium, the basal membrane and the endothelium [15]. Once in the blood circulation they may reach secondary organs [16]. A study recently demonstrated in an overload situation that inhaled CNTs were able to reach the subpleura in mice and were inducing subpleural fibrosis [17]. Thus inhaled particles firstly get in contact with surfactant and body fluids and will interact as coated particles with tissue [13]. In the blood circulation, CNTs encounter approximately 7000 proteins and isoforms [18,19] which can bind to them, as it has been shown in the literature [20-22]. Investigations of these bound components are essential, as it is not the particle itself that defines the biological active identity. Moreover it is a dynamic interplay of associating and dissociating biomolecules [23,24], which is an entity known as the particles "corona". This biomolecule-particle interplay is governed by a large variety of influencing factors from which the very fundamentals are the characteristics of the nano-sized particle itself and the characteristics of the surrounding media.

Among others (like the crystallinity or the shape), the surface functionalization is considered to be one of the most important characteristics of nano-sized particles [25]. By functionalization (i.e. by modifying the surface) a material exhibits new physical, chemical and biological characteristics. To make the surface negatively or positively charged, carboxyl or amino groups can be covalently attached. Characteristic patterns of bound plasma proteins have been shown with carboxyl- and amino-modified polystyrene particles [26,27]. Additionally, it was demonstrated for CNTs that the protein binding was reduced or altered after functionalization [22,28,29]. However, inherent properties of the surrounding medium such as the presence of organic molecules (e.g. proteins) or detergents [25] also strongly determine the binding characteristics and result in new properties of the particle-biomolecule complex. The binding of proteins on a nano-sized particle can change the proteins native conformation [23,30] and may result in the presentation of novel epitopes [30,31]. The new complex triggers (inappropriate) cellular signaling [32,33], initiate protein fibrillation [34], may undergo new transport mechanisms or may be opsonized by the mononuclear phagocytic system [35]. The presence of such opsonins on the particles surface creates a "molecular signature" which may affect the eventual fate of the nano-sized particles in the body [13,36] or have implications on the particles adverse effects [23]. Thus for a detailed understanding of the CNT - cell interaction, a careful assessment of the adsorbed biomolecules has to be included.

The aim of this study was to characterize the binding of biomolecules to different functionalized MWCNTs to simulate their entry into the blood circulation, in a cell free system. From current knowledge, it was not yet considered that inhaled CNTs get in contact with pulmonary surfactant prior to serum proteins. Thus it was of central interest to investigate if the presence of this surfactant alters the protein binding later in the bloodstream and to investigate if the initially bound biomolecules (in particular the surfactant lipids) are exchanged due to dynamic processes.

## Results and discussion

**Figure 1:**
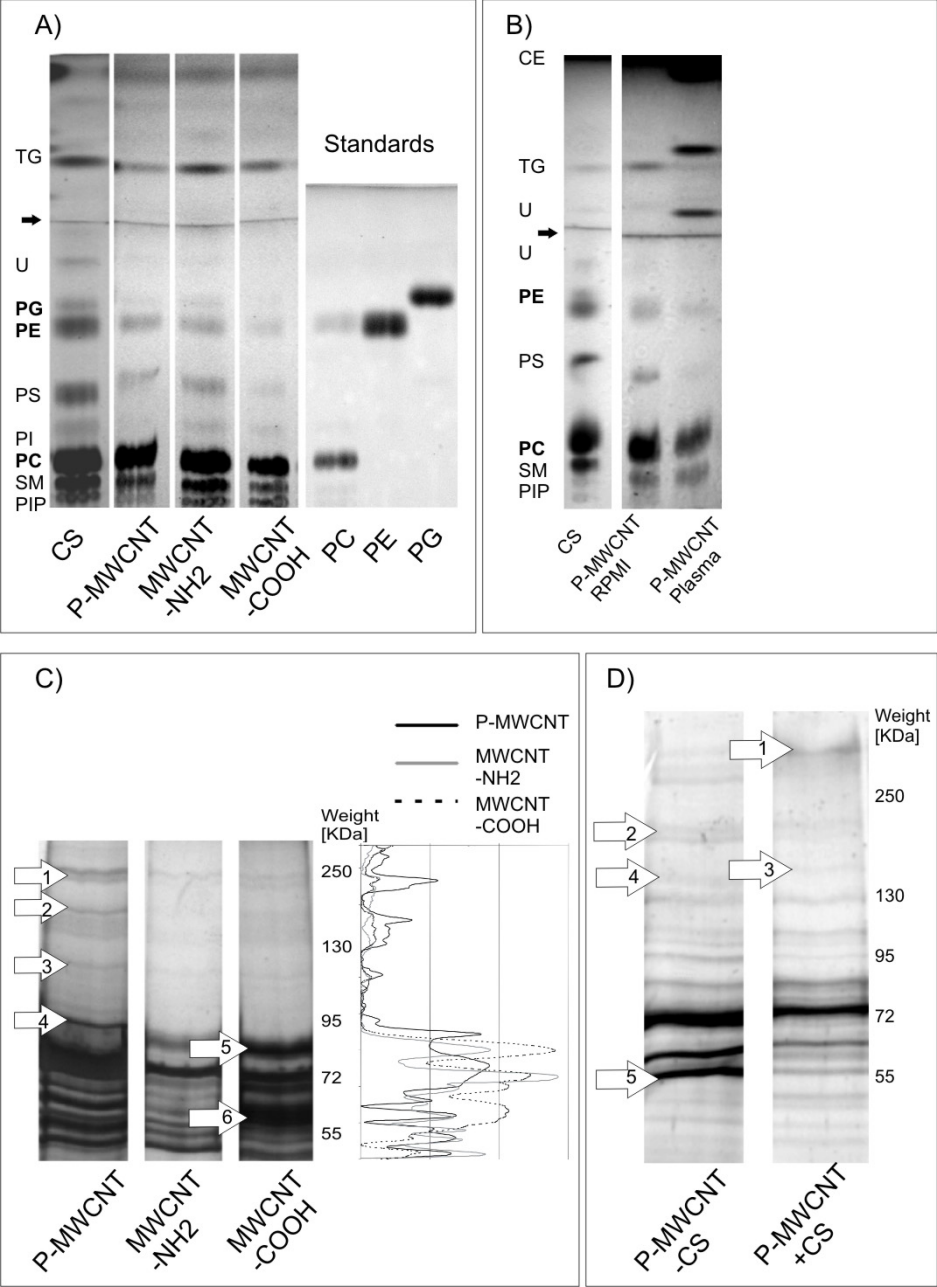
**Identification of lipids and proteins bound to MWCNTs**. **A)**TLC separation of bound lipid components. From left to right: Lipids from pure Curosurf (CS), lipids bound to the P-MWCNT, MWCNT-NH_2_, MWCNT-COOH. Abbreviations for the lipids: TG Triglyceride, PG Phosphatidylglycerol, PE Phosphatidylethanolamie, PS Phosphatidylserine, PI Phosphatidylinositol, PC Phosphatidylcholine, SM Sphingomyelin, PIP Phosphatidylinositolphosphate. Lipid classes were allocated by comparisons to the literature [37,61] and in addition three of the most abundant lipids (Phosphatidylcholine, Phosphatidylethanolamine, Phosphatidylglycerol) were confirmed by the use of standards (lanes 5-7). The arrow points to the front of the first solvent. **B) **Lipids bound to P-MWCNT incubated in Curosurf and post-incubated in Roswell Park Memorial Institute Medium (RPMI) and in blood plasma respectively. RPMI which was used as a control for cell culture medium did not alter the lipid patterns which were obtained by pure Curosurf incubation. The arrow points to the front of the first solvent. **C) **Plasma proteins adsorbed on the different functionalized MWCNTs separated by SDS-PAGE (left part) and quantified by densitometry (right part). 1. Alpha-2-macroglobulin precursor; 2. Complement factor H; 3. Inter-alpha (globulin) inhibitors H1, H2, H4, Complement component 7, Plasminogen; 4. Gelsolin isoform c, Cadherin-5; 5. Coagulation factor XI; 6. Keratin 6A. **D) **Effect of a Curosurf pre-incubation (P-MWCNT+CS) on the protein adsorption pattern. Arrows point to characteristical bands. 1. Apolipoprotein A (precursor), Apolipoprotein B (precursor); 2. Unknown; 3. Ceruloplasmin; 4. Unknown; 5. Fibrinogen beta chain.

**Figure 2:**
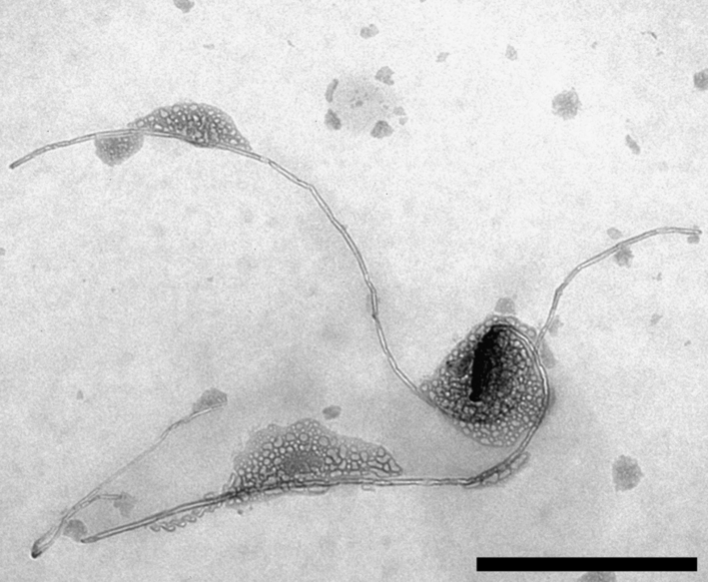
**TEM image of P-MWCNTs which were coated with Curosurf and subsequently washed**. The scale bar is 0.5 μm.

Pristine MWCNTs (P-MWCNTs) and MWCNTs functionalized with positively (-NH_2_) and negatively (-COOH) charged side groups were characterized with different coatings (Table 1). The first coating, which should simulate an initial encounter of MWCNTs with a biological structure in the lung, was investigated by characterizing CNT-bound surfactant lipids. MWCNTs were coated with Curosurf (Chiesi, Parma, Italy), a well characterized natural porcine surfactant preparation [37-39]. The properties and the composition of Curosurf are similar to human pulmonary surfactant and thus it is widely used in the treatment or prophylaxis of the neonatal respiratory distress syndrome [40-42]. By using thin layer chromatography (TLC), it was shown that patterns of MWCNT bound surfactant lipids were identical to the patterns of the complete surfactant (Figure 1A). This finding indicates an unspecific binding, i.e. no influence of the functional groups, which may be explained by the hydrophobic properties of the MWCNTs. The coating of MWCNTs with pulmonary surfactant components was confirmed with transmission electron microscopy (TEM) (Figure 2). It was observed that lipophilic surfactant components foster adhesion among MWCNTs; a phenomenon that was also similarly described in a previous study on carbon black [11]. Such an effect may become more relevant when MWCNTs get in a more hydrophilic environment (as it may happen during a translocation into the hypophase) and remain associated through hydrophobic forces.

**Table 1:** Characterization of MWCNTs.

	P-MWCNT	**MWCNT-NH**_**2**_	MWCNT-COOH
Length [nm]	500 to >2000		

External diameter [nm]	20 - 30		

Specific surface area [m^2^/g] [67]	250-400		

Number of side groups [22] [modifications/1000 nm length]	-	~5000	

Zeta-potential in H_2_O [mV]	-2	+26	-57

Zeta-potential in plasma [mV]	-23	-24	-24

Zeta-potential in Curosurf [mV]	-63	-50	-56

To examine if lipid coatings undergo further dynamic changes, MWCNTs were pre-incubated in Curosurf and subsequently incubated in blood plasma. Figure 1B shows that patterns of bound (surfactant) lipids were clearly altered after subsequent plasma incubation. On the one hand, characteristic lipids from blood (cholesterol ester and triglycerides) were found to bind on MWCNTs and on the other hand the appearance of phosphatidylserine, a lipid from Curosurf, was less pronounced.

If MWCNTs are internalized into cells, the specific lipid coatings may have crucial consequences as the molecular signature of the tube may be recognized more as a biological structure with its distinct functions. In addition to the roles lipids play in surfactant, they are known for numerous other functions. Phosphatidylcholine or phosphatidylinositol for example are well known to be involved in signaling. Only phosphatidylinositol and phosphatidylinositolphosphates regulate the activity of at least a dozen enzymes that control many key cellular functions, including differentiation, metabolism and proliferation [43]. Definitive consequences of a possible translocation of these lipids by CNTs to sites of action are not fully understood and further investigations are needed.

MWCNTs that reach the pulmonary blood circulation can interact with numerous proteins. To investigate if functionalization has a direct influence on the protein patterns, plasma proteins bound to different MWCNTs were identified. Figure 1C shows plasma proteins which were bound to the different functionalized MWCNTs. Six characteristic proteins, which were specific or clearly pronounced for one type of MWCNT, were reproducibly identified after separation by sodium dodecylsulfate polyacrylamide gel electrophoresis (SDS-PAGE). Mass spectrometric (MS) investigations of the protein composition from specific bands revealed further that single gel bands contain high numbers of bound proteins. Nevertheless, characteristic proteins could be assigned to the bands by including the number of detected peptides and excluding proteins from outside the bands weight range ("background"). Thus it was indicated that there were different proteins binding to MWCNTs which were not functionalized (P-MWCNT) compared to both the positive MWCNT-NH_2 _and the negative MWCNT-COOH. Such differences were more pronounced between pristine and functionalized MWCNTs, whereas among functionalized MWCNTs less variability was found. Visual and densitometric (Figure 1C, right section) analyses of the gels showed a noticeable trend for heavier proteins (>100 kDa) on P-MWCNTs compared to functionalized MWCNTs. Hence it can be hypothesized that, at these conditions, surface charge properties only play a minor role in contrast to steric hindrance which prevents larger proteins to bind to functionalized tubes - a phenomenon that is also described in literature [25]. In contrast, smaller proteins may be favored in such situations. Visual analyses were supported by direct mass spectrometric analysis (additional file 1). The alteration in the protein coating from MWCNTs that translocate across the alveolar epithelium into the pulmonary circulation was simulated by pre-coating the tubes with surfactant, followed by incubation in blood plasma. The identification of five characteristic proteins on P-MWCNTs (Figure 1D) demonstrates that the pre-incubation of MWCNTs in surfactant has an influence on the composition of bound plasma proteins. Surprisingly, on P-MWCNTs which were not pre-coated with surfactant, specific proteins were found, which could not be found on pre-coated ones. It can be hypothesized that these proteins were not able to bind to pre-coated MWCNTs due to altered hydrophobic interactions or steric hindrance by the bound lipids. In contrast, proteins which are only present on pre-coated MWCNTs may have two different origins: either these are components of the surfactant itself or they stem from blood plasma and interact specifically with components of the bound surfactant. Phosphatidylethanolamim, for example, is known to build hydrogen bonds to proteins through its ionizable amine group. Moreover for phosphatidylinositol, specific binding to characteristic domains ("Pleckstrin homology or PH domains") of cellular proteins and unspecific binding due to electrostatic interactions are known [43]. Interestingly, less variability depending on the pre-coating was detected in functionalized MWCNTs. This may be due to decreased lipid binding to the functionalized tubes in comparison to P-MWCNTs. Another reason may be that similar steric hindrance is reached either by pre-coating or by functionalization. This would implicate that the surface properties of a functionalized MWCNT are not changed to the same extent by pre-coating as the surface properties of a P-MWCNT.

After identifying a number of specifically bound proteins, their characteristic properties such as structure, function, weight and isoelectric point were assessed. By using this approach, it was possible to relate the functions of bound proteins with the different conditions (MWCNTs functionalization, surfactant pre-coating). Proteins with a large variety of functions were found to be associated with MWCNTs. Interestingly, apolipoproteins of different types were detected in all conditions (additional file 1). These proteins are well known to bind to the majority of nanoparticles [23,31,44]. In a study where high amounts of apolipoprotein A-1 were found on copolymer nanoparticles [31], the affinity to the hydrophopic particles and the curvature of the particle were denoted as important factors. As the MWCNTs used in the current study had diameters similar to lipoprotein particles from blood, the curvature of the MWCNTs could also be a main reason for the increased binding. Apolipoproteins are constituents of lipoproteins and are responsible for the transport of fats. They regulate the lipid metabolism and may be involved in cardiovascular disease risk [45] and amyloidosis [46-48]. Furthermore, apolipoproteins seem to play an important role in the transport of nano-sized particles across the blood-brain barrier (BBB) [49,50] - this could also be true for MWCNTs.

In contrast to the apolipoproteins, the most abundant blood protein albumin was only detected on MWCNTs that were not pre-coated with Curosurf (additional file 1). This indicates reduced binding of Albumin after coating with the lipids. Albumin exhibits a less organized secondary structure upon adsorption onto a hydrophobic surface [51]. By looking at the proteins function, it was shown that albumin which was bound to single-walled carbon nanotubes (SWCNTs) altered the inflammatory response of RAW264.7 macrophages by a reduction of LPS-mediated Cox-2 induction [20]. These indications would imply that bound Curosurf can modulate the CNTs (pro-) inflammatory potential by a reduction of albumin binding.

In addition, the fibrinogen beta chain binding decreased due to Curosurf pre-coating on P-MWCNTs and MWCNT-NH_2 _(Figure 1D and additional file 1). Fibrinogen has a double function: yielding monomers that polymerize into fibrin and acting as a cofactor in platelet aggregation [52]. Interestingly it was shown that the function of fibrinogen to mediate platelet recognition, adhesion, activation, and aggregation was significantly suppressed when it was adsorbed to SWCNTs [53]. In this case we could expect a smaller decrease in platelet aggregation after Curosurf coating.

Another important group of bound proteins are the Complement components which play a key role in the innate and adaptive immune response. Complement components were found on all 3 types of MWCNTs (additional file 1), however the Complement component 7 and the Complement factor H were found preferentially on P-MWCNTs (Figure 1C). An activation of the Complement system by CNTs via the classical and the alternative pathway could be a consequence [54].

Characteristically bound to P-MWCNT was Alpha-2-Macroglobulin (Figure 1C and additional file 1), which is known to inhibit proteinases [52]; the calcium dependent cell adhesion protein Cadherin [55] (Figure 1C); Gelsolin (Figure 1C), an actin-modulating protein which is Calcium-regulated [56]; Plasminogen (Figure 1C) which dissolves (as Plasmin) the fibrin of blood clots and acts as a proteolytic factor in various other processes, such as in remodeling or inflammation [52]; and the inter-alpha (globulin) inhibitors (Figure 1C) which may act as a Hyaluronan carrier or binding protein [52].

Specifically bound to MWCNT-COOH was Keratin 6A (a constituent protein of the intermediate filaments) and the coagulation factor XI (Figure 1C) (involved in the intrinsic pathway of blood coagulation [57]), which was also detected on MWCNT-NH_2_. Ceruloplasmin, which has its main function in the transport of copper [52,58], was only found on P-MWCNTs that were pre-incubated in Curosurf. Numerous further functions can be assigned to bound proteins (additional file 1). Thereby it has to be taken into account that primary protein functions can alter after binding due to conformational change [22,51,59].

It was of great interest to determine if there are structural or functional similarities among proteins which are bound to MWCNTs of one condition (functionalization or Curosurf pre-coating). Thus, the study aimed to identify characteristic regions by a sequence alignment of the proteins' amino acids. These analyses did not identify a common sequence of amino acids within proteins which were bound to MWCNTs in one condition. Also an analysis of the total charge (isoelectric point) of different proteins did not reveal a tendency. Thus various proteins with very distinct structures bind to the three types of MWCNT tested without any identifiable pattern, indicating that MWCNT were able to adsorb proteins in an unspecific manner or not by a single mechanism only.

## Conclusions

**Figure 3:**
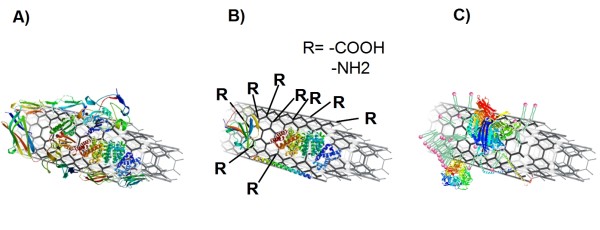
**The binding of blood plasma proteins to MWCNTs under different conditions**. **A) **Blood plasma protein coating on P-MWCNT. **B) **The protein pattern is altered when MWCNTs are functionalized. **C) **A further alteration effect is observed when lipids from surfactant are bound to the MWCNTs. A selection of detected proteins are shown (models adapted from SWISS-MODEL [64-66] and proteinmodelportal.org).

It was shown that lipids and proteins, which are constituents of the air-blood tissue barrier, bind to MWCNTs (Figure 3). Thus the characteristics of MWCNTs change as soon as they are deposited onto the lung surface. Different functionalized MWCNTs are coated similar with lung surfactant lipids which alter the chemical and physical state of the tubes. This first stage coating has several effects on the subsequent blood plasma protein coating (Figure 3C): Firstly, proteins of the surfactant itself bind to the CNTs [21], secondly, bound lipids seem to enable binding of certain plasma proteins and thirdly, other plasma proteins may be sterically hindered to bind by the presence of surfactant components. Like proteins, lipids also undergo dynamic exchange processes and there are strong indications that the composition of bound surfactant lipids is changed, at the latest, when MWCNTs come in contact with blood plasma lipids. With respect to experimental settings, these results point to the importance of considering the surfactant coating in *in vitro *lung models. A way to include this issue is to work with air-liquid interface models [60].

Besides the surfactant pre-coating, the functionalization of the MWCNT was identified as an influencing factor for plasma protein binding (Figure 3B). Thereby the type of functionalization (amino or carboxyl group) seems to play a minor role in contrast to the alteration in hydrophobicity or steric hinderance that results from the functionalization. The latter factor might also be the reason for the increased binding of larger proteins to MWCNTs which were not functionalized. The proteins adsorbed to the surface of the tubes trigger numerous eminent functions, for example they are involved in transport and uptake mechanisms of nano-sized particles or fulfill functions in the immune system. Although consequences on molecular and cellular levels can be estimated, an uncertainty remains as new functions can be expected from bound proteins. With this characterization, a first important step is done and these new findings can be related to toxicology and uptake data with further experiments.

Future focus should be on the possible relationships between the so called "cryptic epitopes" [30] and the cellular effects upon exposure. Hence only by the knowledge of the coronas composition might adverse effects be assessed (the "epitope map" [30]). With such an approach it could be possible to assess adverse effects of nano-objects more easily and to rapidly recommend safety measures to industry.

## Methods

### MWCNTs production and characterization

MWCNTs were synthesized by chemical vapor deposition from Chengdu Carbon Nanomaterials R&D Center (Sichuan, China) and functionalized as previously reported [28]. The Zeta-potential was measured with a Malvern Zetasizer (Malvern Instruments Ltd, Worcestershire, United Kingdom). TEM was performed by a Philips 300 TEM at 60 kV (FEI Company Philips Electron Optics, Zurich, Switzerland).

### Characterization of bound pulmonary surfactant lipids

MWCNTs were dispersed (20 mg/ml) in Curosurf 120 (Chiesi, Parma, Italy), a lipid-based surfactant from pigs. Dispersions were sonicated in a cooled Sonorex RK 156 BH (Bandelin, Berlin, Germany) water bath for 15 minutes. After 24 h of incubation at 37°C, MWCNTs were washed 4 times with phosphate buffered saline (PBS) and centrifuged at a low speed (500 g). Thin layer chromatography (TLC) was performed for the separation of surfactant lipids that were bound to the MWCNTs. The pellet was dispersed in the resolving agent (CHCl_3_/MeOH [2:1]) and 20 μl were pipetted onto a silica gel plate (Merck, Darmstadt, Germany). Pure Curosurf which was diluted (1:10), Phosphatidylcholine, Phosphatidylethanolamine and Phosphatidylglycerol (all from Sigma-Aldrich, Buchs, Switzerland) were dissolved (2 mg/ml) in the resolving agent and used as standards. For improved visualization, two solvents (CHCl_3_/MeOH/HAc/H_2_O [56:33:9:2] and Hexan/Ether/HAc [80:20:1]) were applied [61]. After the chromatographical separation, the plate was placed in a 8%v/v H_3_PO_4 _/10% m/v CuSO_4 _solution and left to develop at 180°C for about 5 min.

### Characterization of bound proteins

MWCNTs in blood plasma (200 μg/ml) were sonicated for 15 min and incubated for 24 h at 37°C. MWCNTs used for a two step coating were pre-coated with Curosurf as described above, washed 3 times with PBS and then the blood plasma was added (200 μg/ml). After 15 min of sonication, MWCNTs were incubated for another 24 h at 37°C and washed 4 times with PBS. Proteins were either directly analyzed by liquid chromatography/tandem mass spectrometry (LC/MS/MS, see below) or detached by adding 6-times concentrated SDS-loading buffer for sodium dodecylsulfate polyacrylamide gel electrophoresis (SDS-PAGE). Proteins were visualized with a Dodeca Silver Stain Kit (Bio-Rad, Reinach, Switzerland) and with a Sypro Ruby Stain Kit (Bio-Rad, Reinach, Switzerland), respectively. Intensities of stained proteins were quantified by the Bio-Rad Quantity One Software on the Fluor-S MultiImager system. Bands that were characteristically found in at least 3 repetitions were cut out and analyzed by LC/MS/MS after Trypsin digestion. All LC/MS/MS samples were analyzed using Mascot (Matrix Science, London, United Kingdom; version Mascot). Mascot was set up to search the NCBInr_20090524 database (selected for Homo sapiens, unknown version, 222717 entries). Scaffold (version Scaffold_2_06_00, Proteome Software Inc., Portland, USA) was used to validate LC/MS/MS based peptide and protein identifications. Peptide identifications were accepted if they could be established at greater than 95.0% probability as specified by the Peptide Prophet algorithm [62]. Protein identifications were accepted if they could be established at greater than 99.9% probability and contained at least 2 identified peptides. Protein probabilities were assigned by the Protein Prophet algorithm [63]. Sequences of characteristically bound amino acids were compared by an online alignment function [52].

## Competing interests

The authors declare that they have no competing interests.

## Authors' contributions

MG participated in the design of the study, carried out the experimental work and drafted the manuscript. BRR was involved in planning the design of the study and made substantial contributions to the analysis and interpretation of the data. HFK and PG made substantial contributions to the analysis and interpretation of the data. BY carried out the synthesis of functionalized MWCNTs. MN accompanied the study as an expert for pulmonary surfactant. All authors read and approved the final manuscript. PW was the project leader, he has intellectually accompanied the experimental work; he has been involved in revising the manuscript critically for important intellectual content and has given final approval of the version to be published. All authors read and approved the final draft.

## Supplementary Material

Additional file 1 **Proteins detected ("X") with direct LC/MS/MS**. Bound proteins which were detected by LC/MS/MS without previous separation by SDS-PAGE. Click here for file
