# Supplementary material for: The adsorption of biomolecules to multi-walled carbon nanotubes is influenced by both pulmonary surfactant lipids and surface chemistry
Source: J Nanobiotechnology. 2010 Dec 15;8:31. doi: 10.1186/1477-3155-8-31 (PMC3012019; doi:10.1186/1477-3155-8-31)
Supplement: Additional file 1 — Proteins detected ("X") with direct LC/MS/MS. Bound proteins which were detected by LC/MS/MS without previous separation by SDS-PAGE. [file 1477-3155-8-31-S1.PDF]

Proteins detected ("X") with direct LC/MS/MS.

	P-MWCNT	P-MWCNT pre-incubated in Curosurf	MWCNT-NH_2	MWCNT-NH_2 pre-incubated in Curosurf	MWCNT-COOH	MWCNT-COOH pre-incubated in Curosurf	Molecular weight (kDa)	Main functions (simplified from UniProt.org)
Apolipoprotein B	X	X		X			516	Major protein constituent of chylomicrons, functions as a recognition signal.
Complement C4-A		X					193	Plays a central role in the activation of the classical pathway of the complement system.
Complement C3	X	X		X	X	X	187	Plays a central role in the activation of the complement system.
Alpha-2-macroglobulin	X						163	Inhibits proteinases.
Inter-alpha-trypsin inhibitor heavy chain H4	X		X	X	X		103	May be involved in acute phase reactions.
Band 3 anion transport protein					X		102	Major integral glycoprotein of the erythrocyte membrane.
Fibrinogen alpha chain	X		X	X	X	X	95	Two main functions: yielding monomers that polymerize into fibrin and acting as a cofactor in platelet aggregation.
Gelsolin			X	X	X		86	Actin-modulating protein.
Serum albumin	X		X		X		69	Main protein of plasma. Regulates the colloidal osmotic pressure of blood.
Histidine-rich glycoprotein				X	X	X	60	The physiological function is not yet known.
Fibrinogen beta chain	X		X			X	56	Two main functions: yielding monomers that polymerize into fibrin and acting as a cofactor in platelet aggregation.
Clusterin	X			X			52	Not yet clear. Has been associated with apoptosis.
Alpha-1-antitrypsin					X	X	47	Inhibitor of serine proteases.
Apolipoprotein A-IV		X					45	Major component of chylomicrons.
Serum paraoxonase/arylesterase 1		X					40	Hydrolytic and antioxidant functions.
Apolipoprotein E	X	X	X	X	X	X	36	Component of chylomicrons. Mediates the binding, internalization, and catabolism of lipoprotein particles.
Apolipoprotein A-I	X	X	X		X		31	Participates in the reverse transport of cholesterol from tissues to the liver.
Serum amyloid A				X			14	Major acute phase reactant. Apolipoprotein of the High-density lipoprotein (HDL) complex.
Apolipoprotein C-II		X					11	Component of the very low density lipoprotein (VLD) fraction in plasma, and activator of several triacylglycerol lipases.
